# In Depth Analysis of the Leading Causes of Maternal Mortality Due to Cesarean Section in Iran 

**Published:** 2017-03

**Authors:** Nasrin Changizi, Golnaz Rezaeizadeh, Leila Janani, Mamak Shariat, Abbas Habibelahi

**Affiliations:** 1 Family Health Institute, Maternal Fetal and Neonatal Research Center, Tehran University of Medical Sciences, Tehran, Iran; 2 Department of Biostatistics, School of Public Health, Iran University of Medical Sciences , Tehran, Iran; Clinical Trial Center (CTC), Tehran University of Medical Sciences, Tehran, Iran; 3 Neonatal Health Office, Ministry of Health, Management and Education of Iran, Tehran, Iran

**Keywords:** Maternal Mortality, Cesarean Section, Risk Factors

## Abstract

**Objective:** Despite the declining trend of maternal mortality (MMR) in Iran between 1990 and 2013, direct causes are still the major reasons for maternal death. One of these direct causes is complications of cesarean section (CS). Since the rate of CS in Iran is quite high (47.9%) and the trend continues to rise, there is an alarming threat of the possibility of increasing MMR in the country as a result of cesarean section complications, especially in repeated cases. In this study, we attempted to determine the indications of CS in reported maternal mortality, with special attention to risk factors predisposing to CS and/or to maternal mortality.

**Materials and methods:** A retrospective study was implemented for the period between March 2009 and March 2012. All nationally reported data regarding maternal death during pregnancy, labor and 42 days after parturition during these 3 years was collected and input to software specially designed for this project. Subsequently, cases of maternal death related to pregnancy termination by cesarean section were selected for analysis.

**Results:** There were 393 cases of maternal death with cesarean section as the termination method. Indications of CS were mostly emergency and repeat and the leading causes of death were postpartum hemorrhage and hypertensive disorders. Most of these deaths occurred in academic hospitals and the most common type of delay was brought about by hospital management, specifically personnel issues.

**Conclusion:** Based on this study, acknowledging CS as a serious health threat endangering every achievement in the maternal health program is the most important policy and efforts should be focused on provision of guidelines for realistic CS indications, standardized CS procedures, and post CS care as well as propagation of training courses in risk management and high risk case-finding protocols.

## Introduction

In the Islamic Republic of Iran (IRI), the maternal mortality rate (MMR) has decreased from 83 deaths per 100,000 live births in 1990 to 23 per 100,000 in 2013 (a 72% reduction in MMR). However, improvements are still required in tracking maternal health status as well as betterment in the quality of maternal care (1).

According to the 10th edition of International Classification of Diseases (ICD), maternal deaths are classified as direct and indirect. Direct maternal deaths are conditions that are specifically due to pregnancy or related complications, while indirect maternal deaths are those resulting from an underlying systemic disease or a disease that is aggravated by pregnancy (1).The leading causes of maternal deaths vary in different geographical regions. In developing countries direct causes (specially hemorrhage) are the leading cause of maternal deaths, but in developed countries leading causes are mainly indirect factors (2).

Though MMR has declined overall from 1990 to 2013 in Iran (1), direct causes of maternal deaths, like postpartum hemorrhage (27%) and preeclampsia (13%) are still the major causes of maternal death (3). Complications of cesarean section (CS) are one of the direct causes of maternal death. There is a general perception that emergency cesarean delivery may increase the possibility of maternal death (4, 5) and, because the CS rate in Iran is very high (47.9%) (6) and increasing (7,8), there is an alarming threat of the possibility of increasing MMR in the country as a result of the effects of CS and its long term complications.

As a result, in this 3-year maternal mortality evaluation project we attempted to determine the indications of CS in reported maternal mortality, with special attention to risk factors predisposing to CS and/or to maternal mortality.

## Materials and methods

***Subjects and protocol*** *:* A retrospective study was performed between March 2009 and March 2012. National Maternal Mortality Surveillance System (NMMSS) software designed using InfoPath was utilized. In the IRI, the NMMSS has been implemented since 2001(9), and data gathering has been performed on paper via written questionnaires since then. The software was designed for gathering all needed data in such a way as to reduce missing data. It was possible to complete the questionnaire both online and offline. 

A software pilot study was performed in 2 maternity hospitals in the Khorasan Razavi province and, based on feedback, problems were resolved and the software was finalized.

In this retrospective study, as we did not intend to perform additional questioning, we defined 0000 as the answer for missing data. This missing data is reflected in the tables that follow. All reported maternal deaths during pregnancy, labor and 42 days after parturition were considered based on the ICD-9 definition. 

This 3 year retrospective survey was performed with the help of at least 50 technicians acquainted with the NMMSS who had been trained to work with the software in 3 different groups at one day workshops. Upon return to their provinces after training, we asked the technicians to input data to the NMMSS during a 2-week period for at least two maternal death files and to advise us as to whether further corrections to the program were necessary. After dealing with their feedback, the project was implemented in the field for 6 months.

Ethics approval (900415917419) for the study was obtained from the Tehran University of Medical Science. All data was confidential.

***Statistical analysis:*** All statistical analyses were performed using the SPSS statistical package version 20 for windows (IBM SPSS Statistics for Windows, Version 20.0. Armonk, NY: IBM Corp).

In this study quantitative variables were reported as mean ± SD, while qualitative variables were reported through frequencies (percentages).

## Results

Over the 3-year project, 896 maternal deaths were registered. Among these deaths 74.4% (n = 664) occurred during or after labor. Table 1 shows the mode of delivery in deaths occurring during or after labor. This study only included maternal deaths occurring after CS (60.9% (n = 393)).

***Maternal deaths after CS*** *:* Demographic characteristics of the mothers are shown in Table 2. With regard to education, 44.9% (n = 168) had less than a high school education while 14.7% (n = 55) were illiterate. Most of these mothers were among the low income population (Annual Income < $3600).

They were mostly between the ages of 18 and 35 years old and were also mostly 2^nd^ and 3^rd^ gravida. Indications of CS were mainly emergency and repeat (Table 3) and the type of anesthesia was primarily general (69.2% (n = 245)) (Table 4). 

**Table 1:** Mode of delivery in deaths occurred during or after labor

Type of childbirth	Direct		Indirect		Unknown		Total death	
	Count	Percent	Count	Percent	Count	Percent	Count	Percent
**NVD**	**168**	**39.5%**	**67**	**35.1%**	**8**	**28.6%**	**243**	**37.7%**
**C/S**	**252**	**59.2%**	**122**	**63.9%**	**19**	**67.9%**	**393**	**60.9%**
**Forceps**	**1**	**0.2%**	**0**	**0.0%**	**0**	**0.0%**	**1**	**0.2%**
**Vacuum**	**4**	**0.9%**	**0**	**0.0%**	**0**	**0.0%**	**4**	**0.6%**
**Pharmacologic** *	**1**	**0.2&**	**2**	**1.0%**	**1**	**3.5%**	**4**	**0.6%**
**No response**	**12**	**-**	**7**	**-**	**0**	**-**	**19**	**-**
**Total**	**438**	**100.0%**	**198**	**100.0%**	**28**	**100.0%**	**664**	**100.0%**

* Induction of labor just by medications.

Most of the deaths occurred in educational hospitals (Table 5). 

The risk factors predisposing to CS and/or to maternal mortality based on indications of CS are shown in Table 6. As can be seen, the leading causes of death were postpartum hemorrhage and hypertensive disorders.

In 60.8% (n = 239) of maternal deaths there had been at least one type of delay of which the most common was related to hospital management, as shown in Table 7. Most delays 61.2% (n = 134) and most errors and cases of neglect 60% (n = 54) occurred in academic hospitals. Errors and cases of neglect occurred in emergency, repeat, elective and perimortem CS deaths at the rates of 57.3% (n = 55), 26% (n = 25), 9.4% (n = 9) and 7.3% (n = 7), respectively. As shown in Table 7, most errors and neglect occurred in deaths that were due to direct causes (24.7% (n = 24) occurred in deaths due to bleeding after delivery and 15.5% (n = 15) in deaths due to preeclampsia and eclampsia).

**Table 2:** Demographic characteristics of dead mothers

**Number of Maternal deaths**	**Total**	**Emergency**	**Repeat**	**Elective**	**Perimortem**
Age group (%)	30.14(6.17)	29.35(6.32)	32.17(4.87)	32.32(7.21)	28.88(6.49)
< 18	6(1.6)	5(2.1)	0(0)	0(0)	1(4.2)
18-34	275(73.3)	184(76.7)	55(66.3)	16(57.1)	20(83.3)
> 35	94(25.1)	51(21.3)	28(33.7)	12(42.9)	3(12.5)
Gestational Age (Week) [Mean (SD)]	35.17(4.98)	34.55(5.42)	36.85(3.50)	36.86(3.77)	32.58(4.55)
Gravida (%)					
1	116(29.5)	97(40.4)	0(0)	9(33.3)	4(17.4)
2-3	173(44)	81(33.8)	55(65.2)	14(51.9)	14(60.9)
4-5	70(17.8)	40(16.7)	21(25)	2(7.4)	5(21.7)
> 6	34(8.7)	22(9.2)	8(9.5)	2(7.4)	0(0)
Education					
Illiterate (%)	55 (14.7)	39(17.3)	7(8.4)	2(8)	2(8.7)
Elementary (%)	104 (27.8)	67(29.3)	22(26.5)	4(16)	8(34.8)
Middle school (%)	64 (17.1)	36(15.9)	17(20.5)	6(24)	3(13)
High school (%)	104 (27.8)	58(25.7)	28(33.7)	8(32)	7(30.4)
University (%)	47 (12.6)	26(11.5)	9(10.8)	5(20)	3(13)
Job					
Housewife (%)	336 (87.0)	210(89.4)	70(83.3)	25(86.2)	20(83.3)
Worker (%)	38 (10.1)	20(8.5)	12(14.3)	3(10.3)	1(4.2)
Student (%)	4 (1.0)	2(0.9)	0(0)	1(3.4)	1(4.2)
Other (%)	7 (1.8)	3(1.3)	2(2.4)	0(0)	2(8.3)
Annual income					
< 3600$ (%)	336(94.1)	216(95.6)	75(91.5)	22(84.6)	23(100)
> 3600$ (%)	21(5.9)	10(4.4)	7(8.5)	4(15.4)	0(0)
Nationality					
Iranian (%)	379 (96.9)	229(95.4)	83(98.8)	29(100)	24(100)
Afghan (%)	9 (2.3)	9(3.8)	0(0)	0(0)	0(0)
Other (%)	3 (0.8)	2(0.8)	1(1.2)	0(0)	0(0)

**Table 3:** Cesarean indications in dead mothers

**Cause of cesarean**	**Direct**		**Indirect**		**Unknown**		**Total death**	
	**Count**	**Percent**	**Count**	**Percent**	**Count**	**Percent**	**Count**	**Percent**
Repeat	53	22.2%	31	25.8%	0	0%	84	22.5%
Elective	20	8.4%	6	5.0%	1	6.7%	27	7.2%
Emergency	151	63.2%	76	63.3%	13	86.6%	240	64.2%
Perimortem	15	6.2%	7	5.9%	1	6.7%	23	6.1%
No response	13	-	2	-	4	-	19	-
Total	252	100.0%	122	100.0%	19	100.0%	393	100.0%

## Discussion

Because of the alarming threat of the possibility of an increase in the MMR in the IRI as a result of the effects of CS and its long term complications, we attempted to determine the indications of CS in reported maternal mortality with special attention to risk factors predisposing to CS in maternal mortality cases using a three-year survey project. Based on this study, the leading causes of cesarean sections among maternal deaths, as in other developing countries (10), were postpartum hemorrhage and hypertensive disorders (direct causes). The rates of other causes of maternal death in our study were also in the range of other developing countries (11).

Socioeconomic status, socioeconomic deprivation and cultural factors are closely associated with maternal death (10) because they result in decreased awareness of mothers about themselves which can bring about delays in recognizing obstetric danger signs, making decisions to seek care, and identifying and reaching a medical facility (type 1 and 2 delays) (10,12). Therefore, policies to increase the level of awareness of mothers can be protective against maternal death (10). However, the most common type of delay in our hospitals was related to hospital management. Maternal deaths and also CS often occur in high risk pregnancies which are usually referred to academic hospitals; therefore, the management of such hospitals should be performed by the most experienced health care providers in the hospital. However, unfortunately this is not the case. In most of our academic hospitals mainly junior obstetrics and gynecology residents are the first line care providers and their delays in understanding situations and making decisions can lead to major problems. Many studies (12), just as ours, have found that delay resulting from hospital management (type 3 delay) was the most common type of delay among the three types of delays that resulted in maternal deaths. 

Based on a systematic review of this third type of delay, the delays are primarily related to human resources (issues related to quality of training/skill and shortages in healthcare personnel) (13). Consequently, health system managers should reconsider their policies about the responsibilities of medical residents and hospital management systems.

Another issue that should be mentioned here is that emboli and thromboembolism rates were higher in repeat CS than in other CS groups. This may be due to higher age and gravidity in this group (Table 2) which are risk factors for these complications (14), so practitioners should pay particular attention to thromboembolism prophylaxis among this group.

## Conclusion

The majority of maternal deaths in developing countries are preventable. In the cases analyzed in this study, reducing direct causes of maternal death can be accomplished by forestalling both delays brought about by weaknesses in hospital management and medical errors, especially in postpartum hemorrhage and preeclampsia-eclampsia.

**Table 4:** Type of Anesthesia in dead mothers

**Type of Anesthesia**	**Direct**		**Indirect**		**Unknown**		**Total death**	
	**Count**	**Percent**	**Count**	**Percent**	**Count**	**Percent**	**Count**	**Percent**
General	172	73.2%	72	61.5%	10	66.7%	254	69.2%
Regional	63	26.8%	45	38.5%	5	33.3%	113	30.8%
No response	17	-	5	-	4	-	26	-
Total	252	100.0%	122	100.0%	19	100.0%	393	100.0%

**Table 5:** Type of hospital administration in dead mothers

**Type of hospital**	**Direct**		**Indirect**		**Unknown**		**Total death**	
	**Count**	**Percent**	**Count**	**Percent**	**Count**	**Percent**	**Count**	**Percent**
GOV Educational	126	53.4%	69	60.0%	13	81.3%	208	56.7%
GOV Treatment	65	27.5%	23	20.0%	2	12.5%	90	24.5%
GOV Other organs	7	3.0%	2	1.7%	0	0.0%	9	2.5%
Private	23	9.7%	9	7.8%	0	0.0%	32	8.7%
Charity	1	0.4%	1	0.9%	1	6.2%	3	0.8%
Social Security	13	5.6%	11	9.6%	0	0.0%	24	6.5%
Azad University	1	0.4%	0	0.0%	0	0.0%	1	0.3%
Total	252	100.0%	122	100.0%	19	100.0%	393	100.0%

Increasing the level of the awareness of expectant mothers can also be protective against maternal death. However, the most important policy that is required is acknowledgment of CS as a serious health threat that has the potential to endanger all advances made in the maternal health program in order that efforts become focused on provision of guidelines for realistic CS indications, standardization of CS procedures and post CS care, and propagation of training courses in risk management and high risk case finding protocols.

**Table 6:** The risk factors predisposing to CS and/or to maternal mortality based on indications of CS

**Cause of Death**	**Total [No. (%)]**	**Emergency**	**Repeat**	**Elective**	**Perimortem**
Direct					
Bleeding before delivery	1 (0.2)	1(0.4)	0(0)	0(0)	0(0)
Bleeding during delivery	11 (2.9)	6(2.5)	4(4.8)	1(3.4)	0(0)
Bleeding after delivery	79 (20.9)	44(18.3)	25(29.8)	10(34.5)	0(0)
Before delivery sepsis	6 (1.6)	3(1.2)	2(2.4)	0(0)	1(4.2)
After delivery sepsis	12 (3.2)	7(2.9)	2(2.4)	3(10.3)	0(0)
Emboli	11 (2.9)	5(2.1)	5(6)	1(3.4)	0(0)
Regional anesthesia complication	3 (0.8)	2(0.8)	1(1.2)	0(0)	0(0)
General anesthesia complication	5 (1.3)	3(1.2)	1(1.2)	1(3.4)	0(0)
Fatty liver	9 (2.4)	9(3.7)	0(0)	0(0)	0(0)
Preeclampsia	38 (10.1)	32(13.3)	2(2.4)	1(3.4)	3(12.5)
Eclampsia	34 (9)	20(8.3)	5(6)	1(3.4)	8(33.3)
Abortion	0(0)	0(0)	0(0)	0(0)	0(0)
Ectopic pregnancy	0(0)	0(0)	0(0)	0(0)	0(0)
Molar pregnancy	0(0)	0(0)	0(0)	0(0)	0(0)
Other	32(8.5)	20(8.3)	6(7.1)	3(10.3)	3(12.5)
All direct	241 (63.8)	152 (63)	53 (60.9)	21(72.1)	15(62.5)
Indirect					
Cardiovascular	31 (8.2)	22(9.1)	6(7.1)	1(3.5)	2(8.3)
HIV	0(0)	0(0)	0(0)	0(0)	0(0)
Diabetes Mellitus	1 (0.3)	1(0.4)	0(0)	0(0)	0(0)
Errors	1 (0.3)	1(0.4)	0(0)	0(0)	0(0)
Bowel perforation	5 (1.3)	3(1.2)	2(2.4)	0(0)	0(0)
Renal diseases	7 (1.9)	5(2.1)	2(2.4)	0(0)	0(0)
Peritonitis	3 (0.8)	2(0.9)	0(0)	1(3.5)	0(0)
Tuberculosis	1 (0.3)	0(0)	1(1.2)	0(0)	0(0)
Chronic HTN	10 (2.6)	7(2.9)	2(2.4)	0(0)	1(4.2)
Thromboemboli	18 (4.7)	11(4.6)	6(7.1)	0(0)	1(4.2)
Other	45(11.8)	24(10)	12(14.3)	5(17.4)	4(16.7)
All indirect	122 (32.2)	76 (31.6)	31 (36.9)	7(24.4)	8(33.4)
Unknown	15 (4)	13(5.4)	0(0)	1(3.5)	1(4.1)
Total	378 (100.2)	241(100)	84(100)	29(100)	24(100)

**Table 7:** Type of delay in dead mothers

**Type of Delay**	**Direct (252)**		**Indirect (122)**		**Unknown (19)**		**Total death (393)**	
	**Count**	**Percent**	**Count**	**Percent**	**Count**	**Percent**	**Count**	**Percent**
Delay	162	64.3%	67	54.9%	10	52.6%	239	60.8%
Delay in decision making	66	26.2%	24	19.7%	2	10.5%	92	23.4%
Delay in referral	47	18.7%	20	16.4%	2	10.5%	69	17.6%
Delay in hospital management	105	41.7%	41	33.6%	4	21.1%	150	38.2%
Errors and neglects	65	25.8%	30	24.6%	2	10.5%	97	24.7%
